# In silico and in vitro identification of secoisolariciresinol as a re-sensitizer of P-glycoprotein-dependent doxorubicin-resistance NCI/ADR-RES cancer cells

**DOI:** 10.7717/peerj.9163

**Published:** 2020-06-10

**Authors:** Mohamed A. Morsy, Azza A.K. El-Sheikh, Ahmed R.N. Ibrahim, Katharigatta N. Venugopala, Mahmoud Kandeel

**Affiliations:** 1 Department of Pharmaceutical Sciences/College of Clinical Pharmacy, King Faisal University, Al-Ahsa, Eastern Region, Saudi Arabia; 2 Department of Pharmacology/Faculty of Medicine, Minia University, El-Minia, Egypt; 3 Basic Health Sciences Department/Faculty of Medicine, Princess Nourah bint Abdulrahman University, Riyadh, Saudi Arabia; 4 Department of Clinical Pharmacy/College of Pharmacy, King Khalid University, Abha, Saudi Arabia; 5 Department of Biochemistry/Faculty of Pharmacy, Minia University, El-Minia, Egypt; 6 Department of Biotechnology and Food Technology, Durban University of Technology, Durban, South Africa; 7 Department of Biomedical Sciences/College of Veterinary Medicine, King Faisal University, Al-Ahsa, Eastern Region, Saudi Arabia; 8 Department of Pharmacology/Faculty of Veterinary Medicine, Kafrelsheikh University, Kafr El-Sheikh, Egypt

**Keywords:** Doxorubicin, Molecular dynamics, P-glycoprotein, Secoisolariciresinol, Rhodamine assay, MTT assay

## Abstract

P-glycoprotein (P-gp) is one of the highly expressed cancer cell efflux transporters that cause the failure of chemotherapy. To reverse P-gp induced multidrug resistance, we employed a flaxseed-derived lignan; secoisolariciresinol (SECO) that acts as an inhibitor of breast cancer resistance protein; another efflux transporter that shares some substrate/inhibitor specificity with P-gp. Molecular dynamics (MD) simulation identified SECO as a possible P-gp inhibitor. Comparing root mean square deviation (RMSD) of P-gp bound with SECO with that bound to its standard inhibitor verapamil showed that fluctuations in RMSD were lower in P-gp bound to SECO demonstrating higher stability of the complex of P-gp with SECO. In addition, the superimposition of P-gp structures after MD simulation showed that the nucleotide-binding domains of P-gp bound to SECO undertook a more central closer position compared with that bound to verapamil. Using rhodamine efflux assay on NCI/ADR-RES cancer cells, SECO was confirmed as a P-gp inhibitor, where cells treated with 25 or 50 µM of SECO showed significantly higher fluorescence intensity compared to control. Using MTT assay, SECO alone showed dose-dependent cytotoxicity, where 25 or 50 µM of SECO caused significantly less NCI/ADR-RES cellular viability compared to control. Furthermore, when 50 µM of SECO was added to doxorubicin (DOX), an anticancer drug, SECO significantly enhanced DOX-induced cytotoxicity compared to DOX alone. The combination index calculated by CompuSyn software indicated synergism between DOX and SECO. Our results suggest SECO as a novel P-gp inhibitor that can re-sensitize cancer cells during DOX chemotherapy.

## Introduction

Cancer is one of the leading causes of death worldwide, especially in developed countries, where nearly 4 million newly diagnosed cancer cases are anticipated in Europe in only the year 2018, with breast cancer the most prevalent (Ferlay et al., 2018). One of the major problems facing the treatment of cancer is multidrug resistance (MDR) caused, at least in part, by the expression of efflux protein pumps that extrude chemotherapeutic drugs outside cancer cells, thus decreasing medication concentration and causing treatment failure (Zhou, Wang & Li, 2018). One of these efflux pumps is P-glycoprotein (P-gp) that was reported in several in vitro and in vivo studies to be expressed in cancer cells, especially those of the breast (Pokharel et al., 2016; Tulsyan, Mittal & Mittal, 2016; Badowska-Kozakiewicz, Sobol & Patera, 2017; Babaer et al., 2018).

P-gp, previously identified as an MDR1 protein, encoded by one of the members of ATP-binding cassette (ABC) genes, namely subfamily B member 1 (ABCB1), is a 170 kDa protein spanning the cell membrane (Yano, Tomono & Ogihara, 2018) having two nucleotide-binding domains (NBDs) and six transmembrane domains (TMDs). NBDs typically bear the transporter ATP-binding site that can bind one or two molecules of ATP, where both NBDs come in contact together in a reverse head-to-tail format, thus enclosing an ATP molecule at the interface of NBDs. The TMDs contains a highly nonspecific substrate-binding site with a substrate translocation path in its center halfway through the cell membrane (Chufan, Sim & Ambudkar, 2015). P-gp adopts several conformational changes with various molecular dynamics (MD) changes (Moradi & Tajkhorshid, 2013) including inward open conformation in the form of a V-shaped structure that opens toward the intracellular side exposing a high affinity substrate-binding site, an outward open conformation in which the substrate is ready to be exported outside the cell, and, in between, two intermediate or transitional steps including inward occluded and outward occluded steps implying the occluded site by a substrate at initial binding and before release, respectively (Szöllősi et al., 2018). The substrate-binding site contains a series of hydrophobic residues that attract a wide range of compounds with high-affinity attractions. During the transition of P-gp from inward open to outward open conformation, these hydrophobic residues change their orientation so their affinity to the substrates decreases to release the substrate to outside the cells (Esser et al., 2017).

The presence of positively charged residues at the portals of P-gp as R355 has a great role in the attraction of negatively charged molecules by electrostatic forces (Marcoux et al., 2013). Hydrophobic force is the major force of binding of standard P-gp inhibitors as verapamil (McCormick, Vogel & Wise, 2015). Therefore, we hypothesize that the design of new P-gp inhibitors should have two conserved features; a hydrophobic core to maintain hydrophobic interactions and stacking with hydrophobic residues at the translocation path, as well as charged hydroxyl groups to aid in the initial electrostatic interaction with charged residues in the attraction site of P-gp. Interestingly, compounds that act as substrates and/or inhibitors of P-gp might share the same effect with other efflux transporters as the breast cancer resistance protein (BCRP; Zhou et al., 2009; Zhang et al., 2018), encoded by the ABCG2 gene. Indeed, both P-gp and BCRP share common substrate specificity, including doxorubicin (DOX), a well-known anticancer drug (Tiwari et al., 2009). It is, thus, logical that both transporters might share the same inhibitors of DOX transport. A lignan derived from flaxseed, namely secoisolariciresinol (SECO), was reported as an inhibitor of BCRP (García-Mateos et al., 2018). The aim of the current study was to prove, using in silico MD studies, that SECO interacts with P-gp by comparing it to verapamil, as well as to confirm this interaction by in vitro studies using cancer cells.

## Materials and Methods

### Software, drugs, and chemicals

Molegro Virtual Docker (MVD) 5.5 software perpetual package license (2012) was purchased from CLC bio (Aarhus, Denmark). All docking studies were run by using MVD. The YASARA Structure software (version 18.4.24) package license (2018) was purchased from YASARA Biosciences GmbH (Vienne, Austria). GraphPad Prism version 5.05 for Windows was purchased from GraphPad Software Inc. (San Diego, CA, USA). Dulbecco’s Modified Eagle Medium (DMEM) was obtained from Thermo Fisher Scientific/Gibco (Waltham, MA, USA). DOX-HCl 10 mg vial was purchased from Pharmacia Italia (Milan, Italy). Verapamil and SECO were purchased from Sigma–Aldrich Co. (St. Louis, MO, USA).

### Protein preparation and docking studies

The Protein Data Bank (PDB) ID 5KOY bound with ATP was used as a template to dock SECO and verapamil. Before docking, the structures were prepared by the addition of missing chains and atoms by using a modeler module implemented in YASARA Structure software. Water and other non-relevant molecules were removed, polar hydrogens were added followed by 3D optimization and energy minimization. Molegro 5.5 software was used in the docking study. Before docking, the structures of the compounds were imported from the PubChem database, desalted and 3D optimized by LigPrep using OPLS2005 force field. The docking grid was generated after template docking. MolDock score was selected and grid resolution was set to 0.3 Å. The template was set by selecting the residues that form the recent resolved P-gp structure 6QEX. Ten poses were generated and ordered by their score. The best pose with the highest score was selected for further analysis. The initial structures were energy minimized, equilibrated and checked for the stability of complexes by running a brief MD simulation for 10 ns (Fig. S1). The brief MD was adopted to assess the binding pattern and the binding of drugs to the P-gp active site. The sites and docking interactions are provided in Fig. S2.

### MD simulation

The YASARA Structure software (version 18.4.24) was used for all MD simulations. The force field was AMBER14, with Lipid14 parameters for non-standard residues. P-gp was placed in a lipid patch (28 Å membrane core) containing phosphatidylcholine and then soaked in water. Initially, the lipid patch was constructed, equilibrated and packed before placing the protein. The membrane extension around protein was set to 30 Å. The protein was temporarily scaled by 0.9 along the *XZ* axes, then lipids with an atom closer than 0.75 Å to protein atom and forming a strong clashing with membrane are deleted. The temporary scaling was removed by a short simulation at 296 K in vacuo. The whole system is immersed in explicit aqueous solvation box. The protein was scaled by 1.02 along the *XZ*-axes every 200 fs, while the membrane was allowed to pack around the protein in ideal geometry. To mimic physiological conditions, counter ions were added to neutralize the system; Na or Cl was added in replacement of water to give a total NaCl concentration of 0.9%. The pH was set at 7.4 as YASARA pKa utility assigns pKa values at selected pH and the bonding orders are assigned and missing hydrogens are added. Initial energy minimization was carried out under relaxed constraints using steepest descent minimization and simulation was continued to 1 ns. The main simulation was then run with particle mesh Ewald electrostatic potential and 8.0 Å cutoff for non-bonded real space forces, a 4 fs time-step, constrained hydrogen atoms, and at constant pressure and temperature (NPT ensemble). All simulation steps were run by a modified preinstalled macro (md_runfastmembrane.mcr) within YASARA package. After the simulation, the average structure was used to calculate root mean square deviation (RMSD) and root mean square fluctuation (RMSF) of each structure model.

### Cellular efflux assay using rhodamine-123

The DOX-resistant NCI/ADR-RES cells were cultured as previously reported (Riehle et al., 2016). To evaluate the transport function of P-gp, briefly, 1 μM of rhodamine-123 was incubated with NCI/ADR-RES cells at 37 °C for 60 min, alone or in the presence of 1, 5, 10, 25, 50 μM of SECO. Verapamil, at a concentration of 10 μM, was used as a positive control for P-gp inhibition. After washing the cells in phosphate-buffered saline, they were lysed. The accumulation of rhodamine-123 intracellularly was estimated using spectrofluorimetry, at excitation and emission wavelengths of 485 and 535 nm, respectively. Results were expressed as a percentage of rhodamine-123 accumulating intracellularly compared to that of the control, based on the following equation: (Sample/Control) × 100.

### Cellular cytotoxicity/viability assay

Using 3-(4,5-dimethylthiazol-2-yl)-2,5-diphenyltetrazolium bromide (MTT), cellular viability of DOX-resistant NCI/ADR-RES cells was performed, where 10^4^ cells/well were re-cultured in 96-well plates for 24 h incubation in DMEM. Cells were treated with either different concentration of SECO alone, or together with 1 µM DOX and incubated for 24 h. Verapamil at 10 µM concentration, either alone or with 1 µM DOX, was used as a positive control. After which, 15 μl of MTT reagent in phosphate-buffered saline at a concentration of 5 mg/ml were added to each well and incubated for 4 h at 37 °C away from light. After the incubation period, 100 μl of DMSO at temperature 37 °C were put in each well and incubated for 10 min to dissolve formazan crystals. Absorbance was then measured at 540 nm via microplate reader. Evaluation of the interaction between DOX and SECO was determined by the combination index method using CompuSyn software (Chou & Martin, 2005).

### Statistical analysis

Results of rhodamine and MTT assays were represented as means ± SEM. Data were subjected to statistical analysis using one-way analysis of variance (ANOVA) followed by post analysis test of Tukey–Kramer, comparing all groups via GraphPad Prism version 5.05. The *P* value was considered significant if less than 0.05.

## Results

### P-gp binding site for SECO

SECO showed comparable scores to those of verapamil, a well-known P-gp inhibitor, where SECO produced a lower MolDock score but with a higher rerank score compared with verapamil (Table 1). In addition, the site of interaction of SECO was partially overlapping with that of verapamil and bears a similar interaction profile of predominant hydrophobic interactions but with improved hydrogen bonding score. This led us to continue experiments to characterize SECO’s P-gp inhibitory potential. SECO displayed stronger binding to the substrate-binding site, by showing higher negative rerank, compared to verapamil.

**Table 1:** Docking parameters of verapamil and secoisolariciresinol (SECO) into P-glycoprotein substrate-binding site.

	Ligand	MolDock score	Rerank score	H-Bond score
Docking into the substrate-binding site	Verapamil	−133.3	−67.9	−2.7
	SECO	−123.2	−96.7	−2.0

### MD comparison of SECO with verapamil as inhibitors of P-gp

**Figure 1:**
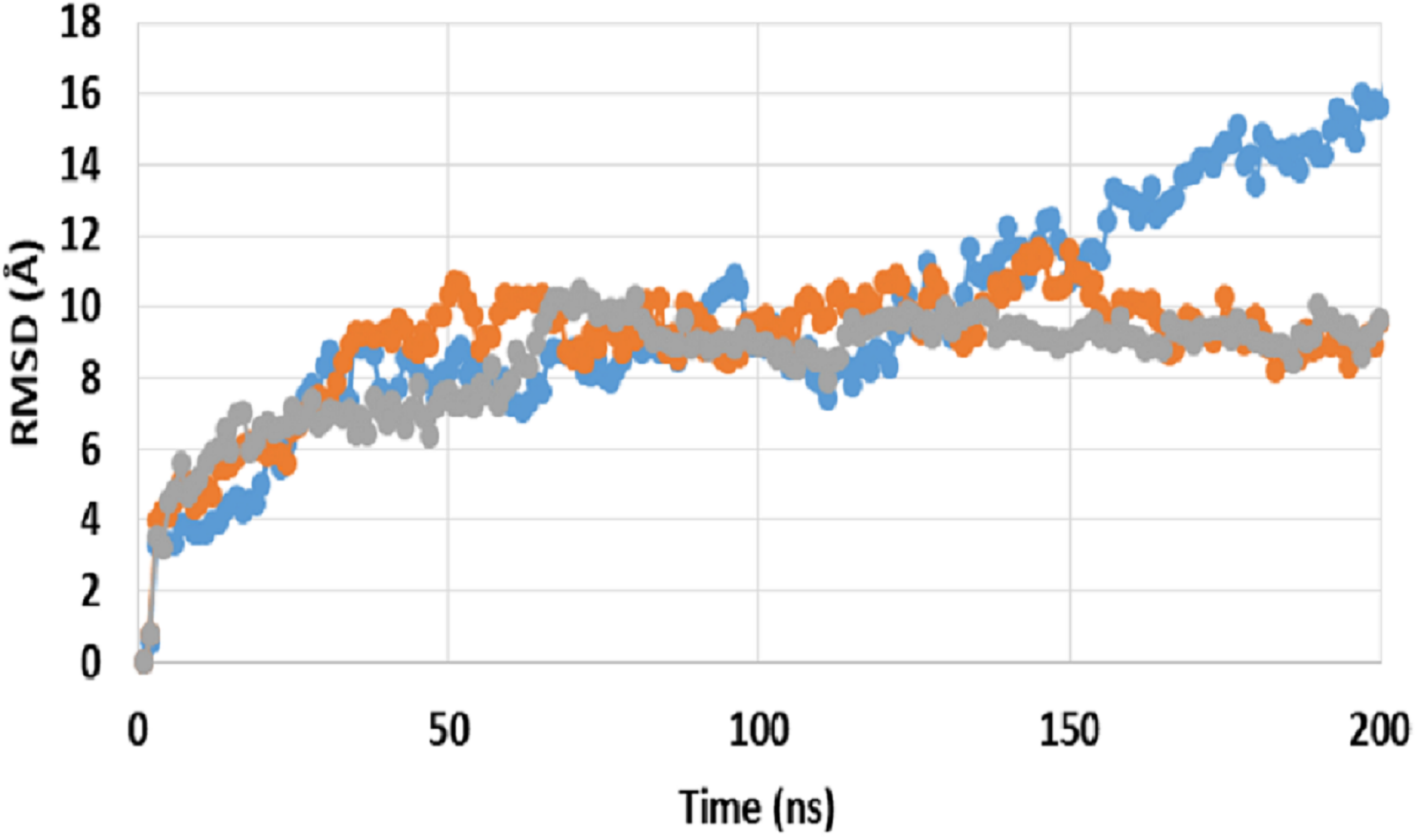
Root mean square deviation (RMSD) of ApoP-gp and P-glycoprotein (P-gp) bound with verapamil or secoisolariciresinol (SECO) structures. The *X*-axis shows the simulation time in nanoseconds (ns) and the *Y*-axis shows RMSDs of the structures during the simulation time. ApoP-gp is the empty form of P-gp without binding to any ligand (blue), verapamil (orange), SECO (grey).

A comparison between RMSD of P-gp bound with SECO (P-gpSECO), P-gp bound to its standard inhibitor verapamil (P-gpVerapamil) and the empty form of P-gp without binding to any ligands (ApoP-gp) has been performed (Fig. 1). P-gpSECO and P-gpVerapamil had more or less similar profiles with slower stabilization until 60 ns and constant low fluctuations in RMSD around 8 Å over the entire recorded simulation. In contrast, ApoP-gp was less stable, showing high fluctuations in RMSD with major drift at 90 ns. Obviously, P-gpVerapamil reached stabilization at an earlier stage at 32 ns compared to P-gpSECO, which showed a gradual increase in RMSD until 62 ns. However, the fluctuations in RMSD were lower in P-gpSECO compared to P-gpVerapamil indicating a more stable complex of P-gp with SECO.

**Figure 2:**
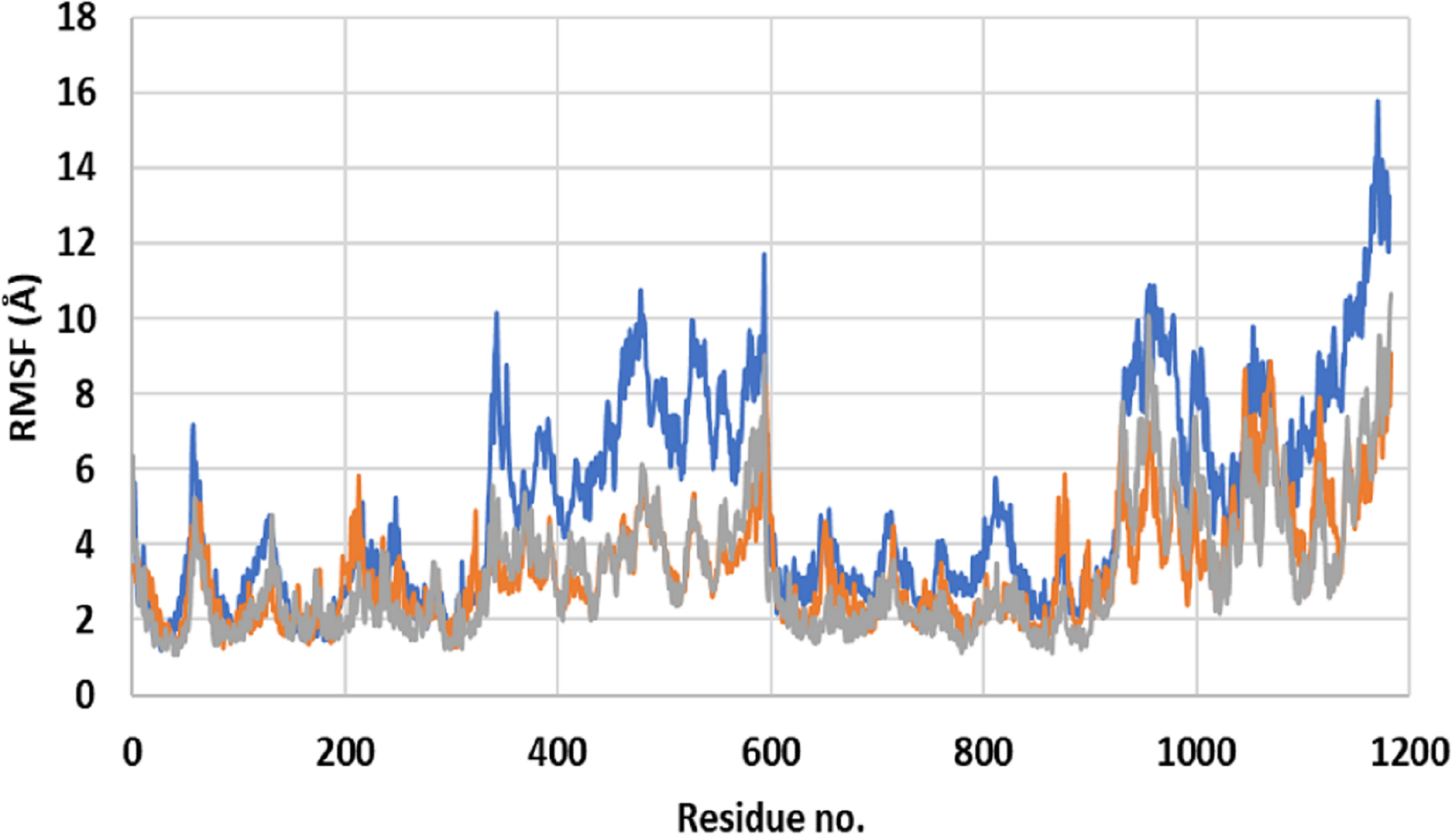
Root mean square fluctuation (RMSF) of residues of ApoP-gp and P-glycoprotein (P-gp) bound with verapamil or secoisolariciresinol (SECO) structures. The *X*-axis shows P-gp residue number and the *Y*-axis shows RMSFs of the residues during the simulation. ApoP-gp is the empty form of P-gp without binding to any ligand (blue), verapamil (orange), SECO (grey).

Close inspection of per-residue RMSF during MD simulation revealed three interesting observations (Fig. 2). The first was the high RMSF of residues in ApoP-gp. In correlation with the whole structure RMSDs during simulation, residues of ApoP-gp were highly fluctuating and indicating continuous movement of ApoP-gp substructures in preparation for substrate recognition. The second observation was the generalized similarity of RMSF of P-gp bound with either verapamil or SECO. The third observation was the lower RMSFs of P-gp bound with verapamil or SECO in several locations on P-gp structure, in comparison with ApoP-gp. This includes the range from S370–T626 and at the C-terminal residues A1085-A1271 (NBDs). Several other residues showed changes in RMSF within 1–3 Å for example, K848–Q918.

**Figure 3:**
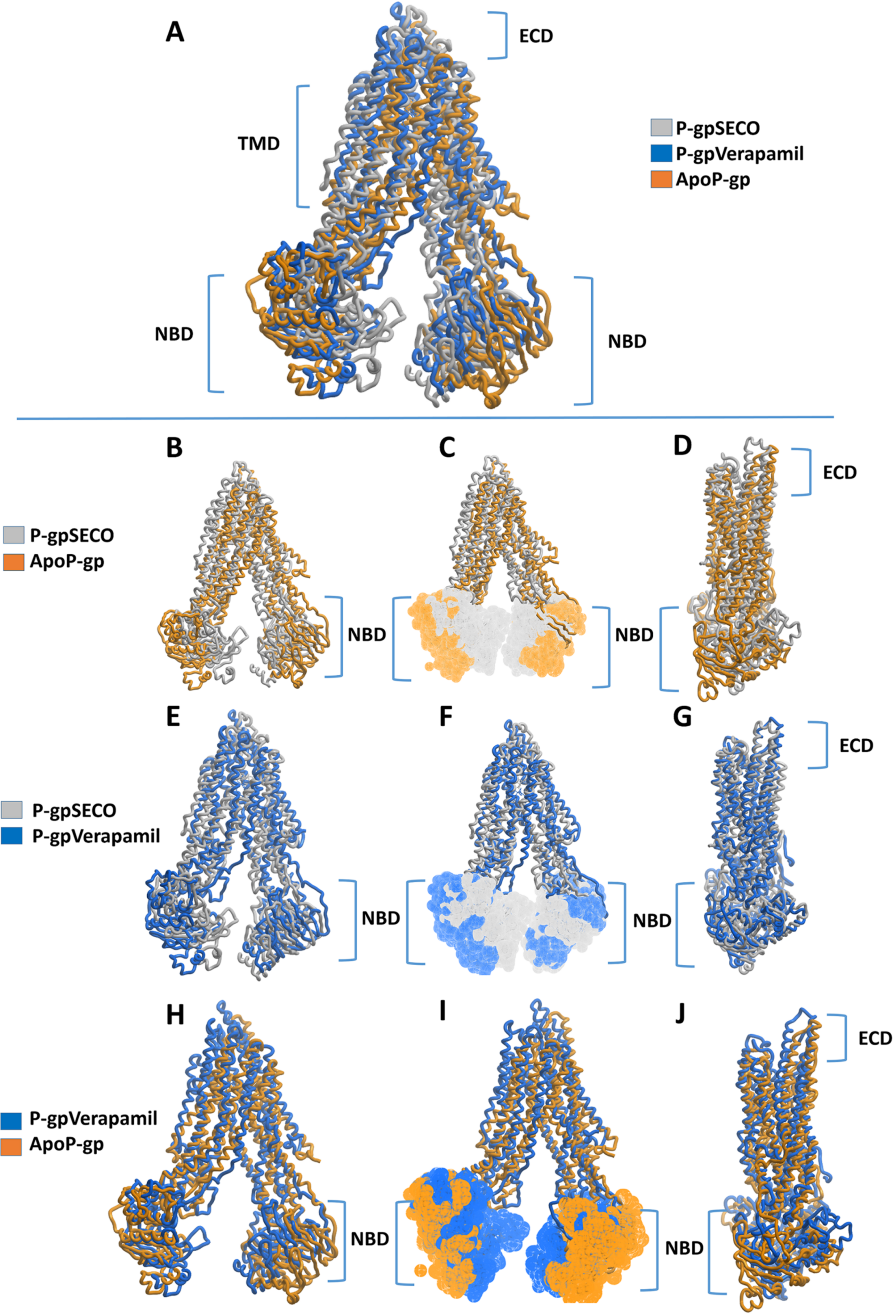
Superimposed P-glycoprotein (P-gp) structures after molecular dynamics (MD) simulation. In (A), three P-gp structures were superimposed including P-gpSECO (grey), P-gpVerapamil (blue) and the empty form of P-gp without binding to any ligand; ApoP-gp (orange). In (B–J), several alignments of two structures were displayed including P-gpSECO and ApoP-gp, P-gpSECO and P-gpVerapamil or P-gpVerapamil and ApoP-gp. For displaying the differences after MD simulation, the structures were represented as thick coil (B, E and H), surface representation of NBDs (C, F and I) or thick coil side view (D, G and J). SECO, secoisolariciresinol; NBD, nucleotide-binding domain.

Superimposition of average structures after MD simulation revealed some differences between SECO-, verapamil-bound P-gp and ApoP-gp (Fig. 3). The position of the two NBDs in relation to each other showed marked differences among the structures, where the distance between NBDs was ApoP-gp > P-gpSECO > P-gpVerapamil (Figs. 3A, 3C, 3F and 3I). In Fig. 3F, the NBDs of P-gpSECO undertook more central closer position (grey color) compared to P-gpVerapamil (blue color), which constituted more peripheral higher distance between NBDs. Similarly, Figs. 3B and 3C explained the closer NBDs of SECO-bound structure (grey color) compared with ApoP-gp (orange color) and Figs. 3H and 3I indicated a closer position of NBDs in verapamil-bound structure compared to ApoP-gp. The side view of P-gp structures superimposition highlights the differences in the extracellular domain (ECD) (Figs. 3D, 3G and 3J). Minor differences were observed between SECO and verapamil-bound structures (Fig. 3G), while P-gp bound with inhibitors showed noticeable changes compared to ApoP-gp (Figs. 3D and 3J).

**Figure 4:**
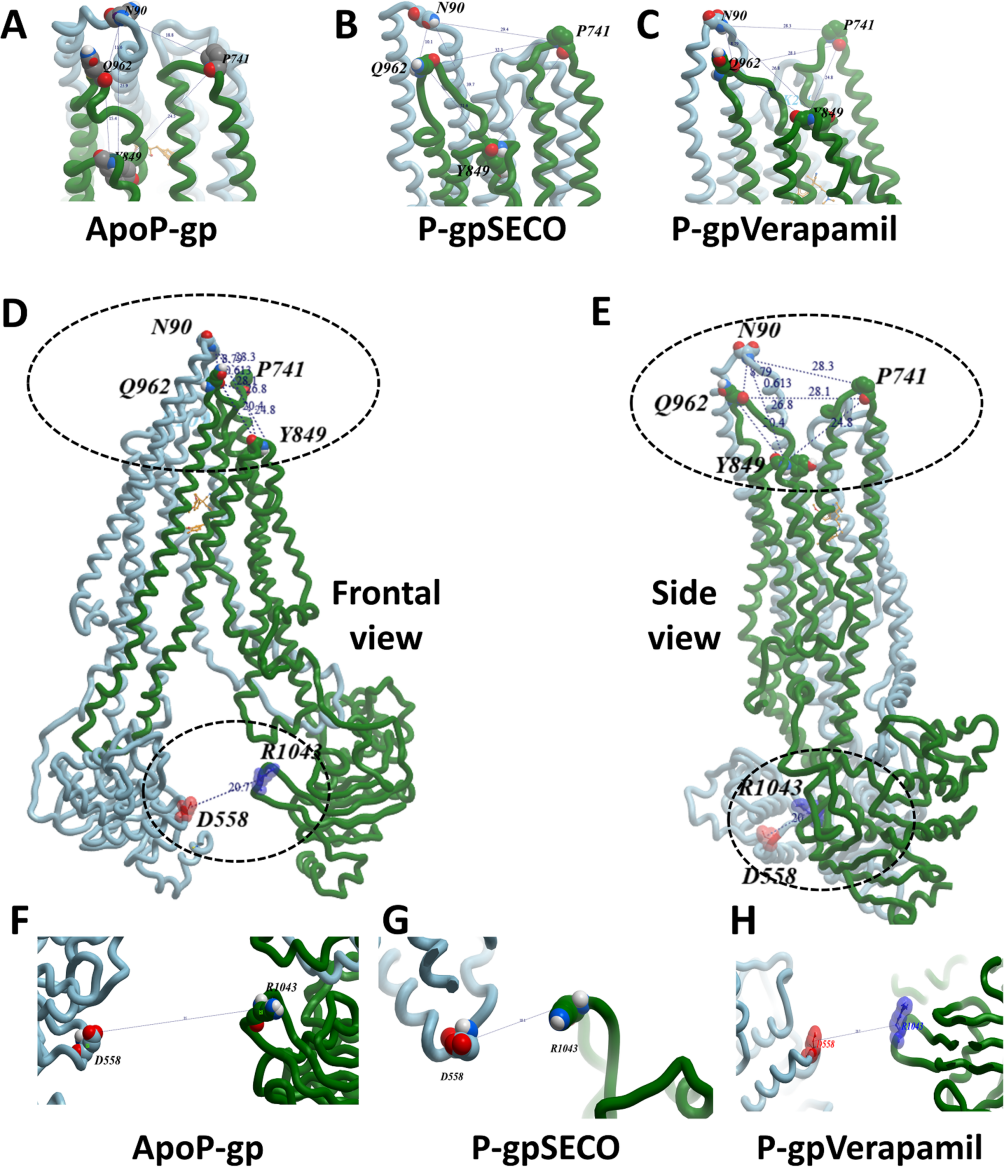
The measured distances between selected residues in nucleotide-binding domains and the extracellular loops of P-glycoprotein (P-gp). Four residues (N90, P741, Y849, and Q962) were selected in extracellular loops (A–E). The distance between cysteines of the two Walker domains and R1043-D558 in nucleotide-binding domains (F–H). ApoP-gp, the empty form of P-gp without binding to any ligand; SECO, secoisolariciresinol.

To deeply assess the structural changes of P-pg bound with inhibitors, a set of key indicator residues were selected at the NBDs and extracellular loops (ECLs) connecting TMD1-2, TMD3-4, and TMD5-6. At NBDs, the distance between D558 and R1043 and the two cysteines of Walker A domain was selected as an indicator of NDBs translocation (Table 2; Fig. 4). In ECL, four residues were selected including N90 (at the top of the hairpin formed between TMD1 and TMD2), P741 (at the top of the hairpin formed between TMD1 and TMD2 on the second monomer of P-gp), Y849 (at the top of the hairpin formed between TMD3 and TMD4) and Q962 (at the top of the hairpin formed between TMD5 and TMD6) (Figs. 4A–4E). The largest space difference was between N90 and P741 in P-gpSECO indicating partial movement in the two P-gp subunits, followed by P-gpVerapamil and lastly by Apo-Pgp, which did not show noticeable differences. In NBDs, the distance between cysteines of the two Walker domains and R1043–D558 (Figs. 4F–4H) was the highest in Apo-P-gp followed by P-gpVerapamil, then P-gpSECO (Table 2). Taking the distance between N90 and P741 as the standard for comparing the P-gp structural changes, the distance was in the following order: ApoP-gp > P-gpVerapamil > P-gpSECO.

**Table 2:** The measured distance between selected residues in NBDs and ECLs.

Position	Residue	Measured distance (Å)		
		P-gpSECO	P-gpVerapamil	ApoP-gp
NBDs	R1043-D558	18.15	20.7	35
	C427-C1070	21.8	27.8	37
TM3-TM4	G187-G226	13.9	14.5	16.2
ECL	P741-N90	18.77	28.33	32.26
	Q962-N90	11.63	10.15	8.79
	I848-N90	25.95	26.77	29.37
	P741-Y849	24.52	24.82	26
	Q962-Y849	15.35	20.35	21.58

**Note:**

NBD, nucleotide-binding domain; ECL, extracellular loop; P-gp, P-glycoprotein; SECO, secoisolariciresinol; ApoP-gp, empty form of P-glycoprotein without binding to any ligand; TM, transmembrane.

**Figure 5:**
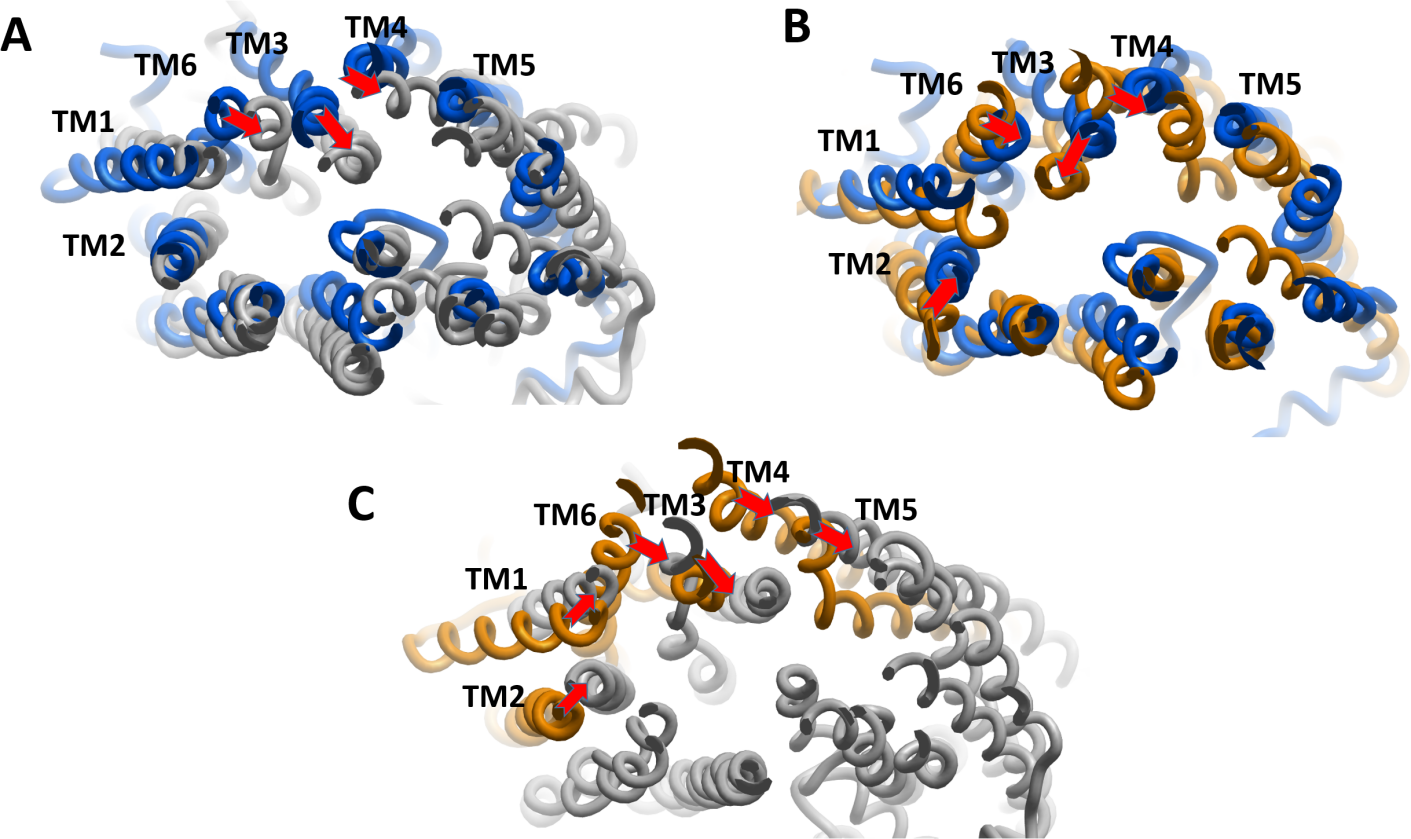
The superimposition of P-glycoprotein (P-gp) structures post molecular dynamics showing the changes in transmembrane (TM) helices. (A) Superimposition of P-gpVerapamil (blue) and ApoP-gp (grey). (B) Superimposition of P-gpVerapamil (blue) and P-gpSECO (brown). (C) Superimposition of P-gpSECO (grey) and ApoP-gp (brown). ApoP-gp: the empty form of P-gp without binding to any ligand; SECO: secoisolariciresinol.

We estimated the distance of displacement of verapamil and SECO between its position at the start of the experiment and after 200 ns of simulation. Verapamil showed 13.5 Å displacement from its initial site of docking (Fig. S3). In contrast, SECO showed lower displacement by showing 8.13 Å (Fig. S4). This demonstrated that SECO was bound more properly and with higher binding efficiency to P-gp compared to verapamil. In addition, the position of TM helices after MD for P-gpSECO showed inward placement of TM1, TM3, TM4 and TM6 into the cavity formed for the substrate binding (Fig. 5). This seemed like a trial from P-gp to displace the ligand from its active site. The inward placement of TMs seemed more prominent with SECO and to a lesser extent with verapamil as deduced from superimposition of P-gpVerapamil and ApoP-gp (Fig. 5A), P-gpVerapamil and P-gpSECO (Fig. 5B) and P-gpSECO and ApoP-gp (Fig. 5C). There was asymmetric changes in P-gp TMs movement with SECO. One monomer of P-gp seemed highly reactive with TMs movements while the other seemed highly fitted with ApoP-gp with little or no changes in the superimposed structure (Fig. 5C).

Secondary structure contents of the whole P-gp system under different conditions, as well as the potential interactions of SECO with TM6, were investigated (Table 3) and showed that, in comparison with ApoP-gp, SECO and verapamil induced higher coil % on the expense of helical content. In addition, TM6 was more disorganized in ApoP-gp. The helical content was increased in P-gpVerapamil structure than ApoP-gp, however, surprisingly, P-gpSECO showed higher helical content (80%) compared to P-gpVerapamil. Ligand interaction analysis in this study revealed a lack of interactions of verapamil as well as SECO with TM6.

**Table 3:** Secondary structure contents (%) of P-glycoprotein and TM6.

		P-gpSECO	P-gpVerapamil	ApoP-gp
Whole structure	Helix	52.2	52.1	56
	Sheet	9.9	9.5	9.5
	Turn	9.8	9.4	9.7
	Coil	24.8	25.9	23.2
	3–10 helix	3.3	3.1	1.6
TM6	Helix	80	52	25
	Sheet	0	0	0
	Turn	0	16	50
	Coil	20	32	25
	3–10 helix	0	0	0

**Note:**

ApoP-gp, empty form of P-glycoprotein without binding to any ligand; SECO, secoisolariciresinol; TM, transmembrane.

### Effect of SECO on rhodamine-123 efflux of DOX-resistant NCI/ADR-RES cells

**Figure 6:**
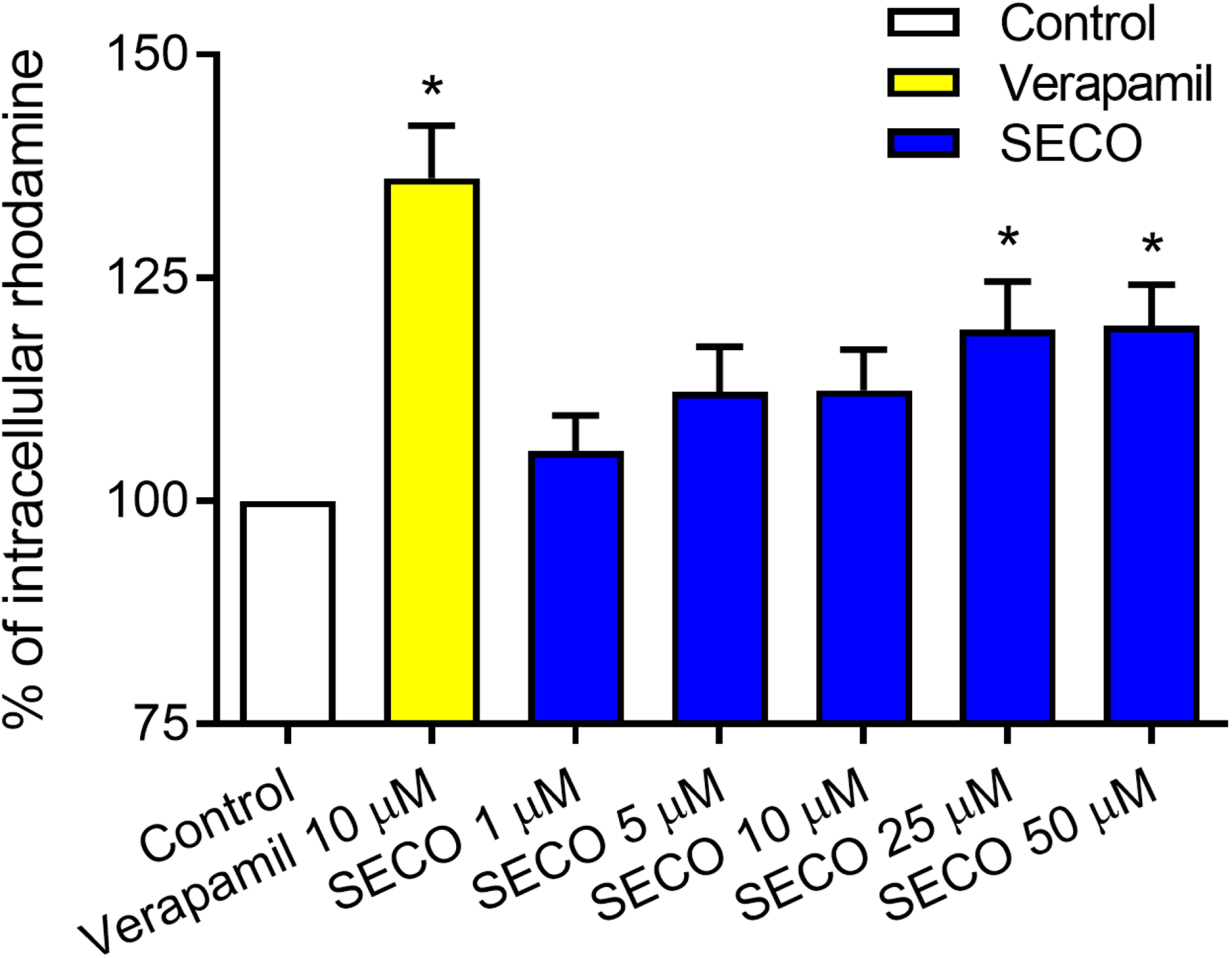
Rhodamine-123 assay of secoisolariciresinol (SECO) in NCI/ADR-RES cells. Data are means ± SEM (*n* = 8), evaluated as percentage according to the following equation: (Sample/Control) × 100. When *P* < 0.05, results were considered statistically significant. *Significant in comparison with control (100%).

To evaluate SECO inhibitory effect on P-gp in vitro, rhodamine-123 efflux assay was used employing P-gp expressed in DOX-resistant NCI/ADR-RES cells and using verapamil 10 μM as a positive control. Various concentrations of SECO were applied (1, 5, 10, 25 and 50 μM). Dose-dependent inhibitory effect of SECO on rhodamine-123 efflux was observed, with significant intracellular accumulation of rhodamine-123 in cells incubated with 25 or 50 μM SECO compared to control cells (Fig. 6).

### In vitro effect of SECO on cellular cytotoxicity

**Figure 7:**
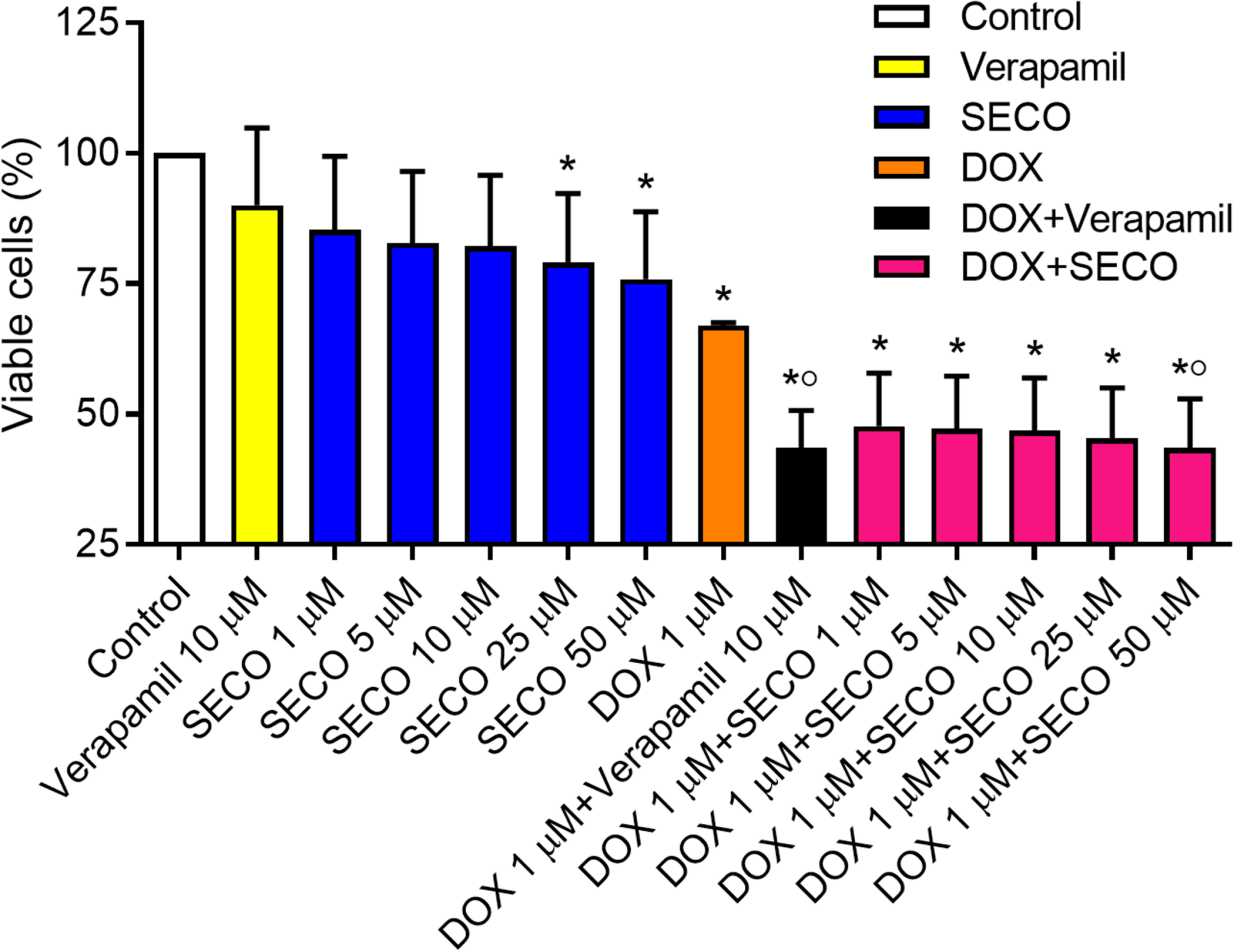
Effect of secoisolariciresinol (SECO) with or without doxorubicin (DOX) on cellular viability. Using MTT assay, SECO, in indicated concentrations, with or without 1 μM of DOX, was incubated with NCI/ADR-RES cells. Data are means ± SEM (*n* = 8). When *P* < 0.05, results were considered statistically significant. *Significant compared to negative control, °Significant compared to positive control (DOX).

The MTT assay was used to assess cellular cytotoxicity. SECO was incubated with NCI/ADR-RES cells in a range of concentrations (1, 5, 10, 25 and 50 μM) in the presence or absence of 1 μM of DOX. Verapamil with or without DOX was used to verify the experiment. Our results showed that DOX alone caused a significant decrease in cellular viability in comparison with negative control (Fig. 7). Administration of DOX with SECO, especially at higher concentration (50 μM) caused a significant increase in cellular cytotoxicity compared to DOX alone. Interestingly, SECO itself at 25 and 50 μM concentrations, without the addition of DOX, showed a significant increase in cellular cytotoxicity compared to control. The combination index (CI) determined by CompuSyn software indicated that at fraction affected (*f*_a_) = 0.5 or higher, the calculated CI is predominantly less than 1. This indicates the synergistic effects when combining SECO with DOX.

## Discussion

Flaxseed has been previously implicated in the prevention and treatment of different types of cancer, including breast and colorectal cancers (Calado et al., 2018; DeLuca, Garcia-Villatoro & Allred, 2018). One of the active ingredients derived from flaxseed is the phenylpropanoid lignan; SECO, which is a metabolite of SECO diglucoside, and has itself anticancer properties (Di et al., 2018). In the current study, SECO has been identified through in silico MD as a potential inhibitor of P-gp; an efflux transporter highly expressed in cancer cells, which was confirmed by in vitro studies performed on NCI/ADR-RES cancer cells.

We have recently performed in silico molecular docking studies of several naturally occurring compounds against human P-gp (Morsy et al., 2018). After the binding of P-gp to any of its substrates, P-gp undergoes dynamic changes in its structure (Zhang et al., 2014). The initial recognition of P-gp to its substrates is thought to be driven by binding electrostatic interacting hotspots on P-gp. In the current experiment, SECO showed preferable and stronger binding score to the substrate-binding site and bearing highly similar computational and MD profile to verapamil. The presence of hydroxyl groups in SECO might have contributed to its inhibitory properties by electrostatic attractions with the substrate-binding site of P-gp.

There are several models describing the catalytic functions of P-gp including nucleotide occlusion (Sauna & Ambudkar, 2007; Siarheyeva, Liu & Sharom, 2010), ATP switch (Higgins & Linton, 2004), processive clamp (Janas et al., 2003), alternating sites (Senior, Al-Shawi & Urbatsch, 1995) and constant contact (Jones & George, 2009). In all cases, NBDs interactions were the forerunner of all P-gp functions by different compilation of events within NBDs and TMDs. The transporter without binding to any substrate (ApoP-gp) seemed to be more dynamically active than when its structures were bound with inhibitors. The ApoP-gp structure showed high RMSDs and continuous drifts even after 60 ns of MD. Interestingly, we found that the intra-cytoplasmic parts of ApoP-gp were more movable allowing wider space for trapping the intra-cyctoplasmic substrates. In addition, we found that the wider space between NBDs was associated with P-gp’s inward open conformation, which is in line with previous reports confirming that the wide separation between NBDs is a characteristic feature of eukaryotic transporter (Jin et al., 2012; Moradi & Tajkhorshid, 2013; Li, Jaimes & Aller, 2014).

In the current study, after 200 ns simulation, the orientation of NBDs was not optimal and the selected residues R1043 and D558 were based on the search for the nearest residues in both NBDs. In addition, the distance between cysteines in Walker domains was used to account for more stable loops positions. Taking into account that the ATP signature region (GSSGCGKS) and Walker A motifs (LSGGQKQ) should complement each other and bind together during NBDs interaction (Xu, Seelig & Bernèche, 2017), these regions were still distant from each other and did not come in close apposition, indicating the effect of SECO and verapamil in preventing NBDs proper interaction process and failure to complete the P-gp necessary structural changes for substrates translocation. The distance between NBDs in its ApoP-gp form was more than 20 Å and the space between the two Walker A motifs in NBDS was not less than 16 Å (Qu & Sharom, 2001; Hellmich et al., 2012). The association between NBDs was affected by the presence of inhibitors bound with the substrate-binding site. The improper association between the signature and Walker A motif in NBDs might have been a result of the effect of the inhibitor on P-gp physiological function. Our results were consistent with previous reports implying an inconsistent association between NBDs in the presence or absence of ATP (O’Mara & Mark, 2014; Condic-Jurkic et al., 2018). After examination of several structures inter-NBDs’ distance distribution, the association of ATP sites from the two interacting NBDs was asymmetric (Condic-Jurkic et al., 2018).

Interactions with TM6 of P-gp is an important drive of attenuation of ATP hydrolysis and a marker for P-gp inhibition (Storm et al., 2008). It was previously noted that hydroxyl radicals can interact with TM6 of P-gp leading to hydroxyl-induced damage due to cleavage of TM6, which might result in deleterious effects on P-gp function (Khosravian et al., 2016). Since SECO contains four reactive hydroxyl groups, we investigated the potential interactions of SECO and TM6. Our results showed that there were no covalent interactions of SECO with TM6, confirming the general safety of SECO. Ironically, P-gpVerapamil showed multiple hydrophobic interactions with TM6 residues. In contrast, SECO only showed hydrophobic interactions with TM12, which is constantly associated with TM6 in P-gp activity (Loo & Clarke, 1999). After the MD simulation, the difference in helical content of TM6 was therefore associated with the interaction of inhibitor with it. The high helical content and rigidity of TM6 with SECO was due to lack of interaction with TM6, while verapamil binding was associated with TM6 flexibility and altered secondary structure content due to multiple hydrophobic interactions, which was consistent with previous studies reporting the predominance of hydrophobic nonpolar interactions of verapamil with P-gp (McCormick, Vogel & Wise, 2015).

After the strong highlight of SECO as an inhibitor of P-gp efflux transporter by in silico studies, in vitro experiments were done for confirmation and showed that SECO at a dose of 25 or 50 μM inhibited P-gp induced efflux of rhodamine-123 and increased its intracellular accumulation in NCI/ADR-RES cancer cells. This is the first study suggesting SECO as a P-gp inhibitor.

In the current study, SECO not only potentiated DOX-induced cytotoxicity in NCI/ADR-RES cancer cells, which is expected due to P-gp inhibition, but also had on its own cellular cytotoxic effect when incubated with the cells for 24 h at a concentration of 25 or 50 μM. Previous studies suggested the cytotoxic effects of SECO in human breast cancer T47D cell line and in mice bearing tumor (Ezzat et al., 2018) as well as in metastatic breast cancer cell lines (Di et al., 2018). On the other hand, SECO was reported to protect normal cells against cytotoxic agents, as it was suggested to protect non-malignant lung cells against radiation injury (Velalopoulou et al., 2015), to prevent apoptosis of myocardial cells caused by oxidative stress (Huang et al., 2018), and to decrease asbestos-induced cytotoxicity of macrophages (Pietrofesa et al., 2018). Such an adaptogenic property; being toxic to cancer cells and protective to normal cells, presents SECO as a potentially safe inhibitor of P-gp mediated chemotherapeutic resistance. It is noteworthy that P-gp is also expressed in normal sanctuary tissues and contributes to the pharmacokinetics of various xenobiotics. Still, the clinical use of flaxseed ingredients, including SECO, was indeed reported as safe in healthy old-aged volunteers (Alcorn et al., 2017; Di et al., 2017). Nevertheless, whether or not 25 and 50 µM SECO is therapeutically attainable will require further evaluation to unravel the dose of SECO needed for the clinical effect.

## Conclusions

The current study presents SECO as a potential novel P-gp inhibitor, with favorable interaction and safe profile. SECO has four hydroxyl groups that may interact with P-gp substrate-binding site occluding its translation channel and cause inhibition of P-gp activity, without interaction or damaging effect on TM6. Based on MD, less inhibitory potential of SECO compared to verapamil is compensated by the lack of interaction with TM6, the higher movement of TMDs toward the inner cavity with asymmetric TMDs movements, and with lower hydrophobic interactions with P-gp. SECO manifested anticancer drug potentiation of DOX and possessed on its own cytotoxic properties in cancer cells.

## Supplemental Information

10.7717/peerj.9163/supp-1 Supplemental Information 1 The chemical structures of secoisolariciresinol (SECO) and verapamil as well as the position of docked SECO (or verapamil) into the substrate-binding site of P-glycoprotein.

10.7717/peerj.9163/supp-2 Supplemental Information 2 The sites and docking interactions of secoisolariciresinol (SECO) and verapamil with P-glycoprotein.

10.7717/peerj.9163/supp-3 Supplemental Information 3 Movement of verapamil through P-glycoprotein.

10.7717/peerj.9163/supp-4 Supplemental Information 4 Movement of secoisolariciresinol through P-glycoprotein.

10.7717/peerj.9163/supp-5 Supplemental Information 5 Secoisolariciresinol data of rhodamine-123 and MTT assays.

10.7717/peerj.9163/supp-6 Supplemental Information 6 The initial structure without any modifications.

10.7717/peerj.9163/supp-7 Supplemental Information 7 The optimized structure with correcting the missing bonds and atoms, water removed and side chains inspected and optimized. The hydrogens were added and the structure was energy minimized.

10.7717/peerj.9163/supp-8 Supplemental Information 8 CompuSyn calculated Fa and CI.

## Additional Information and Declarations

### Data Availability

The following information was supplied regarding data availability: The raw data are available in the Supplemental Files.
