# Supplementary material for: In silico and in vitro identification of secoisolariciresinol as a re-sensitizer of P-glycoprotein-dependent doxorubicin-resistance NCI/ADR-RES cancer cells
Source: PeerJ. 2020 Jun 10;8:e9163. doi: 10.7717/peerj.9163 (PMC7293189; doi:10.7717/peerj.9163)
Supplement: Supplemental Information 1 [file peerj-08-9163-s001.pdf]

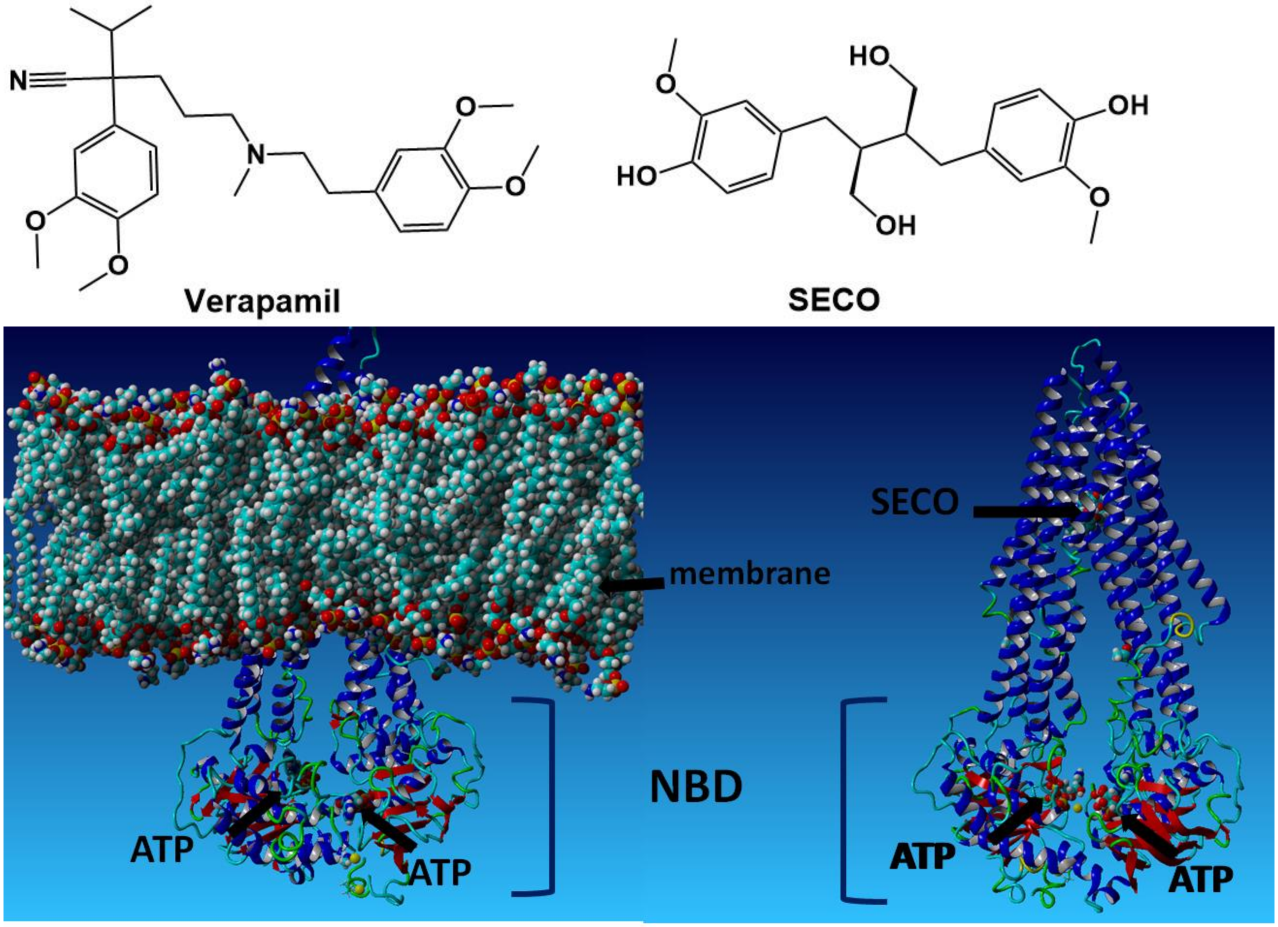

Supplementary Fig. 1 The chemical structures of secoisolariciresinol (SECO) and verapamil as well as the position of docked SECO (or verapamil) into the substrate-binding site of P-glycoprotein. The whole structure was placed in equilibrated membrane patch and used in molecular dynamics experiment.
