# Supplementary figures and images for: In silico and in vitro identification of secoisolariciresinol as a re-sensitizer of P-glycoprotein-dependent doxorubicin-resistance NCI/ADR-RES cancer cells
Source: PeerJ. 2020 Jun 10;8:e9163. doi: 10.7717/peerj.9163 (PMC7293189; doi:10.7717/peerj.9163)

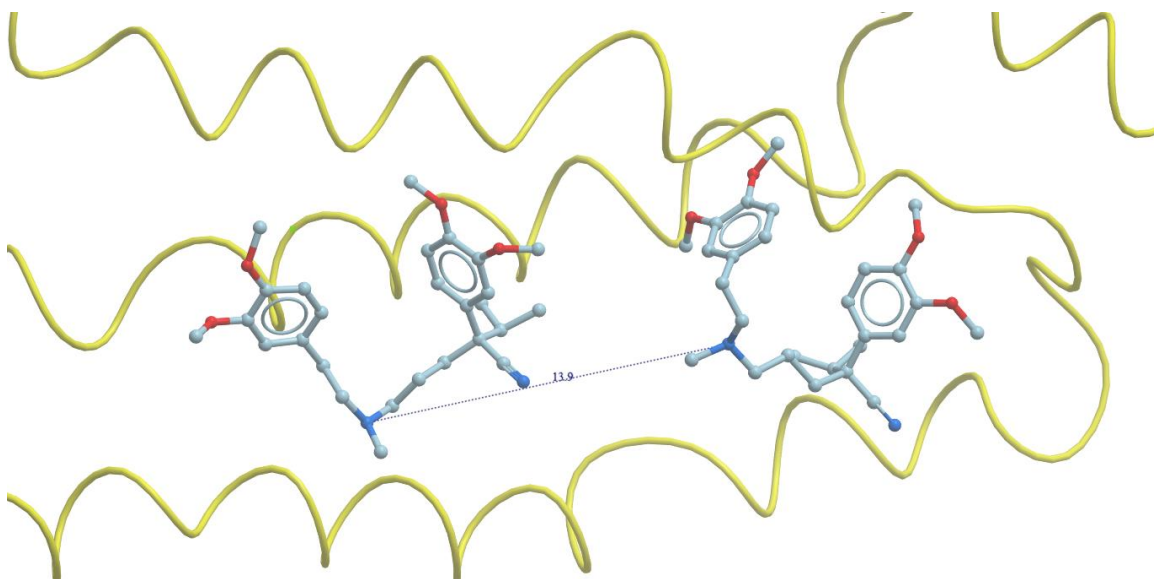

**Supplementary Fig. 3 Movement of verapamil through P-glycoprotein.**

Supplement: Supplemental Information 3 [file peerj-08-9163-s003.pdf]

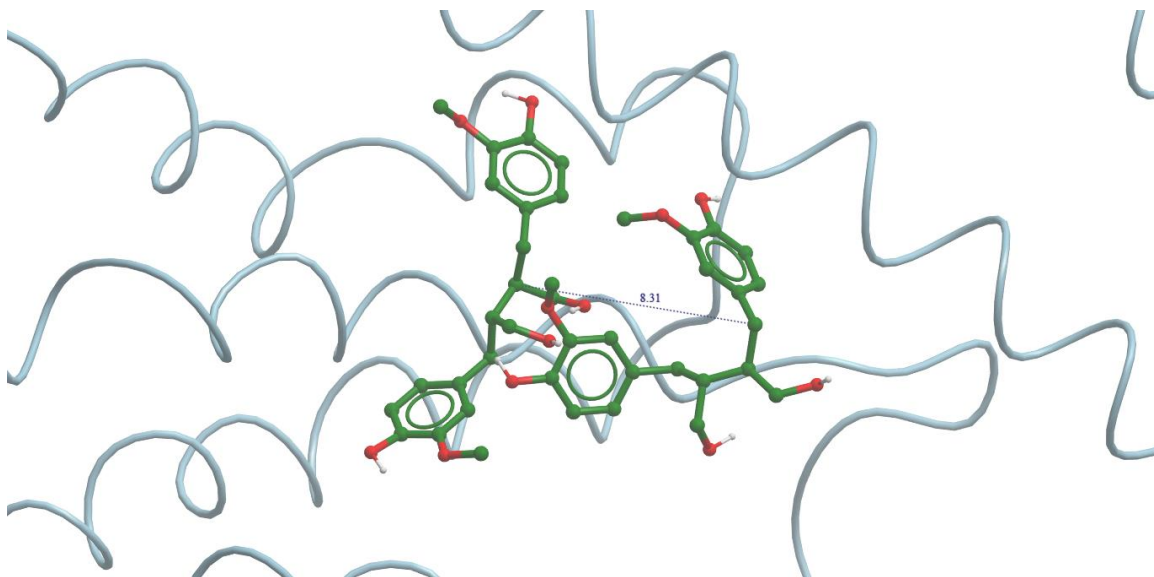

**Supplementary Fig. 4. Movement of secoisolariciresinol through P-glycoprotein.**

Supplement: Supplemental Information 4 [file peerj-08-9163-s004.pdf]
